# Designing Next-Generation Drug-like Molecules for Medicinal Applications

**DOI:** 10.3390/molecules28041860

**Published:** 2023-02-16

**Authors:** Imtiaz Khan, Sumera Zaib

**Affiliations:** 1 Department of Chemistry and Manchester, Institute of Biotechnology, The University of Manchester, 131 Princess Street, Manchester M1 7DN, UK; 2 Department of Basic and Applied Chemistry, Faculty of Science and Technology, University of Central Punjab, Lahore 54590, Pakistan

The development of new drugs/drug candidates for medical treatment remains an exciting but challenging process as only a limited number of synthetic compounds fit well into the discovery and development process after multiple experiments and screening for their preclinical properties. Over the years, this continuous demand has been fueled by the use of organic/synthetic chemistry protocols that deliver new molecules or improve the existing toolbox diversifying libraries of pharmacophores of medicinal interest. The application of new methodologies, particularly employing green and sustainable commercial feedstock chemicals for the discovery and development of biological therapeutics, opens up new avenues of research. In parallel, the discovery and development of new organic molecules have always proved effective in designing drugs, while overcoming critical challenges to the pharmaceutical industry and providing innovative solutions towards commercialized medicines.

This Special Issue aims to provide a far-reaching overview of the most recent developments in synthetic methodologies as well as medicinal chemistry applications of small molecule inhibitors. This Special Issue encompasses fourteen original research articles and three authoritative reviews covering exciting developments in the design strategies of new drugs/drug molecules, structure–activity relationships, in vitro and in silico analyses, and pharmacokinetic properties. These articles not only summarize the recent developments in different perspectives of drug development but also present a wealth of information and possible structural leads to explore new drug inhibitors against various targets of medicinal importance. Herein, we briefly summarize the contributions reported in this Special Issue.

Chang and co-workers summarized the phytochemistry, ethnopharmacological uses, biological activities, and therapeutic applications of *Cassia obtusifolia* L., a member of the Leguminosae family. The ethnomedicinal importance of *C. obtusifolia* indicates that the whole plant, roots, seeds, leaves, stem bark, pods, and fruits are used for the treatment of various health issues. The pharmacological features of the plant include- antimicrobial, anti-inflammatory, antidiabetic, antioxidant, anticancer, neuroprotective, hepatoprotective, immune-modulatory, larvicidal, anti-Alzheimer’s, and anti-Parkinson’s properties [1].

El-Gamal and co-workers reviewed the structure, location, ligands, and functions of HER4 kinase, which play a key role in the normal physiological functions of body systems such as cardiovascular, nervous, and endocrine systems. They have also summarized the relationship of HER4 with the development of various cancers such as colorectal, lung, gastric, prostate, bladder, ovarian, breast, pancreatic, brain, endometrial, melanoma, osteosarcoma, and hepatocellular carcinoma. They also presented a concise summary of selective HER4 inhibitors developed between 2016 and 2022. These classes include quinazoline, quinoline, imidazo-pyrimidine, pyrrolo-pyrimidine, and imidazo-thiazole compounds [2].

In the subsequent study, El-Gamal and co-workers reviewed the pyrazole-based kinase inhibitors developed between 2011 and 2020. Pyrazole is a well-known nitrogen-containing five-membered heterocycle with a proven track-record of a diverse pharmacological importance. Among many other biological functions, pyrazole derivatives have shown their potential as kinase inhibitors that play a crucial role in various cancers. The reported pyrazole-containing compounds have displayed inhibitory effects against various kinases, including Akt, ALK, Aurora, Bcr-Abl, CDK, Chk2, EGFR, ERK/MEK, FGFR, IRAK4, ITK, JAK, JNK, LRRK, Lsrk, MAPK14, PDK4, Pim, RAF, ROS1, Src, and VEGFR [3].

Barakat and co-workers developed a concise library of spirooxindole derivatives through a one-pot three-component approach. α,β-unsaturated ketones, substituted isatin, and (2*S*)-octahydro-1*H*-indole-2-carboxylic acid were coupled together in methanol under reflux conditions. The target compounds were obtained in high regio- and diastereoselectivity. The synthetic compounds were evaluated for their antiproliferative activity against four cancer cell lines, including prostate PC3, cervical HeLa, and triple-negative breast cancer (MCF-7 and MDA-MB231) using an MTT assay. Molecular docking analysis was also performed to rationalize the key binding interactions and results, which demonstrated that the active compounds accommodated well in the binding pocket of MDM2 [4].

Kim and co-workers reported a series of pyridylpyrazole derivatives and evaluated their potential to inhibit lipopolysaccharide (LPS)-induced prostaglandin E2 (PGE2) and the production of nitric oxide (NO) in RAW264.7 macrophages. A multistep synthetic route was established using commercial starting materials to obtain the target compounds. Some of the tested compounds exerted a stronger inhibitory effect on the production of PGE2 than over NO [5].

Khan and co-workers demonstrated a facile multistep synthetic approach to access quinoline-thiosemicarbazone derivatives. A molecular hybridization strategy was used to combine quinoline and thiosemicarbazone pharmacophores in one unit. The in vitro cholinesterase inhibitory assessment of the tested derivatives revealed that several compounds can act as potential drug candidates against Alzheimer’s disease. Kinetics studies were performed, presenting the competitive mode of inhibition. Molecular docking analysis further revealed the conspicuous role of vital binding interactions in the active pocket of cholinesterases, whereas the ADMET profile suggested the safe and druggable properties of quinoline-thiosemicarbazone hybrids [6].

In another study, Khan and co-workers prepared *α*-mangostin (MGN)-loaded nanosponges using a quasi-emulsion solvent evaporation method and investigated their potential to treat type 2 diabetes mellitus. The nanosponges were characterized through FTIR spectroscopy, differential scanning calorimetry (DSC), and scanning electron microscopy (SEM). Various physicochemical properties, including the zeta potential, entrapment efficiency, hydrodynamic diameter, and drug-release properties were also tested. The in vitro *α*-glucosidase inhibition potential demonstrated appreciable results, which were reinforced with in vivo studies [7].

Choi and co-workers reported the anti-diabetic potential of naturally occurring hesperetin and their glycosylated derivatives using protein tyrosine phosphatase 1B (PTP1B). The acquired results were promising and PTP1B inhibition was found to be dependent on the nature, position, and number of sugar moieties in the flavonoid structure. Molecular docking analysis showed a significant binding interaction with key amino acid residues in the PTP1B allosteric site cavity [8].

Anwer and co-workers demonstrated the preparation of four different baricitinib (BTB)-loaded lipids (stearin)-polymer hybrid nanoparticles using a single-step nanoprecipitation method. The nanoparticles were characterized through various physicochemical parameters. The in vitro release and in vivo pharmacokinetic studies in rats revealed the enhanced bioavailability of BTB formulations [9].

A computational approach was designed and utilized by Bajorath and co-workers to explore covalent kinase inhibitors by combining fragment- and structure-based screening with deep generative modeling learning. The approach was exemplified with the design of Bruton’s tyrosine kinase (BTK) inhibitors, which are employed as a major drug target for the treatment of inflammatory diseases and leukemia [10].

Al-Harrasi and co-workers designed and synthesized a small library of 1,3,4-oxadiazole compounds that contain a phenylalanine amino acid. The structures were fully established through various spectroscopic methods. The carbonic anhydrase inhibitory profile was examined and the acquired in vitro efficacy was remarkable, which was further validated with computational modeling analysis [11].

Ul-Haq and co-workers performed in silico screening (virtual screening) for the identification of small-molecule inhibitors of *Streptococcus mutans* glycosyltransferases. They have developed various pharmacophore models which were validated with multiple datasets. Several hits were identified, showing a high binding affinity, and hydrophobic and electrostatic interactions with vital amino acid residues. The identified hits exhibited remarkable antibiofilm activity [12].

Csuk and co-workers synthesized a selection of rhodamine B or rhodamine 101-conjugated pentacyclic triterpenoic amides. Piperazine or homopiperazine were successfully coupled with different acids. The cytotoxic activity of the synthetic derivatives was evaluated using SRB assays. The in vitro results demonstrated that the piperazinyl and homopiperazinyl amides are more cytotoxic than their parent acids or acetylated congeners. Moreover, rhodamine 101-conjugated homopiperazinyl amide showed good results against ovarian cancer cells [13].

Metwaly and co-workers applied an in silico approach for the identification of the best SARS-CoV-2 nsp16-nsp10 2’-*o*-methyltransferase (2’OMTase) inhibitor among several FDA-approved drugs. They have utilized molecular fingerprints, structure similarity analysis, molecular modeling, and MD simulations to identify and support the results [14].

In the search of new VEGFR-2 inhibitors, Eissa and co-workers designed and synthesized several nicotinamide compounds. Anti-proliferative activity results against human cancer cell lines (HCT-116 and HepG2) showed significant promise and excellent VEGFR-2 inhibitory activity. Furthermore, immunomodulatory effects against TNF-*α* and IL-6 were also examined. In silico molecular docking and dynamics simulation demonstrated the high affinity to the target resides and the ADMET profile indicated the acceptable drug-likeness properties [15].

Carradori and co-workers reported coumarin-based amino acyl and (pseudo)-dipeptidyl derivatives. The in vitro dual inhibitory potential against membrane-bound and cytosolic human carbonic anhydrases (hCAs) and monoamine oxidases (hMAOs) as anticancer agents was evaluated. The synthetic compounds exhibited a nanomolar inhibition efficacy. Computational studies shed light on the interesting features required for the inhibitory profiles as well as the isoform selectivity [16].

Jain and co-workers reported a series of ring-modified histidine-containing short cationic peptides. The anticryptococcal activity was evaluated and the acquired results revealed promising antifungal activities against *C. neoformans*. The SEM and TEM analyses indicated the involvement of the cell disruption mechanism [17].

In summary, the current Special Issue encompasses both synthetic and natural inhibitors of various drug targets. The synthetic classes include pyrazole, pyridylpyrazole, oxadiazole, amides, spirooxindole, quinoline-thiosemicarbazone, and coumarin derivatives. The conjugation of amino acids with other biologically active pharmacophores as well as the synthesis of short peptides for medicinal purposes have been achieved. The use of nanotechnology to address the burgeoning health complications have also been illustrated. Various drug delivery approaches highlighted the sustained drug release and enhanced bioavailability of nanoformulations. Moreover, in silico approaches were successfully exploited not only for the identification of potential inhibitors but also for the elucidation of a high affinity to the target residues, thus strengthening the in vitro assay results. Overall, the presented research in this Special Issue demonstrates a significant advancement towards achieving the next-generation drug-like molecules for medicinal applications. 

## References

[1] Ali M.Y., Park S., Chang M. (2021). Phytochemistry, Ethnopharmacological Uses, Biological Activities, and Therapeutic Applications of *Cassia obtusifolia* L.: A Comprehensive Review. Molecules.

[2] El-Gamal M.I., Mewafi N.H., Abdelmotteleb N.E., Emara M.A., Tarazi H., Sbenati R.M., Madkour M.M., Zaraei S.-O., Shahin A.I., Anbar H.S. (2021). A Review of HER4 (ErbB4) Kinase, Its Impact on Cancer, and Its Inhibitors. Molecules.

[3] El-Gamal M.I., Zaraei S.-O., Madkour M.M., Anbar H.S. (2022). Evaluation of Substituted Pyrazole-Based Kinase Inhibitors in One Decade (2011–2020): Current Status and Future Prospects. Molecules.

[4] Al-Majid A.M., Ali M., Islam M.S., Alshahrani S., Alamary A.S., Yousuf S., Choudhary M.I., Barakat A. (2021). Stereoselective Synthesis of the Di-Spirooxindole Analogs Based Oxindole and Cyclohexanone Moieties as Potential Anticancer Agents. Molecules.

[5] Gamal El-Din M.M., El-Gamal M.I., Kwon Y.-D., Kim S.-Y., Han H.-S., Park S.-E., Oh C.-H., Lee K.-T., Kim H.-K. (2021). Evaluation of the Inhibitory Effects of Pyridylpyrazole Derivatives on LPS-Induced PGE2 Productions and Nitric Oxide in Murine RAW 264.7 Macrophages. Molecules.

[6] Zaib S., Munir R., Younas M.T., Kausar N., Ibrar A., Aqsa S., Shahid N., Asif T.T., Alsaab H.O., Khan I. (2021). Hybrid Quinoline-Thiosemicarbazone Therapeutics as a New Treatment Opportunity for Alzheimer’s Disease-Synthesis, In Vitro Cholinesterase Inhibitory Potential and Computational Modeling Analysis. Molecules.

[7] Usman F., Shah H.S., Zaib S., Manee S., Mudassir J., Khan A., Batiha G.E.-S., Abualnaja K.M., Alhashmialameer D., Khan I. (2021). Fabrication and Biological Assessment of Antidiabetic *α*-Mangostin Loaded Nanosponges: In Vitro, In Vivo, and In Silico Studies. Molecules.

[8] Ali M.Y., Jannat S., Jung H.-A., Choi J.-S. (2021). Structural Bases for Hesperetin Derivatives: Inhibition of Protein Tyrosine Phosphatase 1B, Kinetics Mechanism and Molecular Docking Study. Molecules.

[9] Anwer M.K., Ali E.A., Iqbal M., Ahmed M.M., Aldawsari M.F., Saqr A.A., Ansari M.N., Aboudzadeh M.A. (2022). Development of Sustained Release Baricitinib Loaded Lipid-Polymer Hybrid Nanoparticles with Improved Oral Bioavailability. Molecules.

[10] Yoshimori A., Miljković F., Bajorath J. (2022). Approach for the Design of Covalent Protein Kinase Inhibitors via Focused Deep Generative Modeling. Molecules.

[11] Rafiq K., Ur Rehman N., Halim S.A., Khan M., Khan A., Al-Harrasi A. (2022). Design, Synthesis and Molecular Docking Study of Novel 3-Phenyl-Alanine-Based Oxadiazole Analogues as Potent Carbonic Anhydrase II Inhibitors. Molecules.

[12] Atta L., Khalil R., Khan K.M., Zehra M., Saleem F., Nur-e-Alam M., Ul-Haq Z. (2022). Virtual Screening, Synthesis and Biological Evaluation of *Streptococcus mutans* Mediated Biofilm Inhibitors. Molecules.

[13] Heise N.V., Major D., Hoenke S., Kozubek M., Serbian I., Csuk R. (2022). Rhodamine 101 Conjugates of Triterpenoic Amides Are of Comparable Cytotoxicity as Their Rhodamine B Analogs. Molecules.

[14] Eissa I.H., Alesawy M.S., Saleh A.M., Elkaeed E.B., Alsfouk B.A., El-Attar A.-A.M.M., Metwaly A.M. (2022). Ligand and Structure-Based In Silico Determination of the Most Promising SARS-CoV-2 nsp16-nsp10 2′-*o*-Methyltransferase Complex Inhibitors among 3009 FDA Approved Drugs. Molecules.

[15] Yousef R.G., Eldehna W.M., Elwan A., Abdelaziz A.S., Mehany A.B.M., Gobaara I.M.M., Alsfouk B.A., Elkaeed E.B., Metwaly A.M., Eissa I.H. (2022). Design, Synthesis, In Silico and In Vitro Studies of New Immunomodulatory Anticancer Nicotinamide Derivatives Targeting VEGFR-2. Molecules.

[16] Agamennone M., Fantacuzzi M., Carradori S., Petzer A., Petzer J.P., Angeli A., Supuran C.T., Luisi G. (2022). Coumarin-Based Dual Inhibitors of Human Carbonic Anhydrases and Monoamine Oxidases Featuring Amino Acyl and (Pseudo)-Dipeptidyl Appendages: In Vitro and Computational Studies. Molecules.

[17] Sharma K., Aaghaz S., Maurya I.K., Singh S., Rudramurthy S.M., Kumar V., Tikoo K., Jain R. (2023). Ring-Modified Histidine-Containing Cationic Short Peptides Exhibit Anticryptococcal Activity by Cellular Disruption. Molecules.

